# Supplementary material for: Special scattering regimes for conical all-dielectric nanoparticles
Source: Sci Rep. 2022 Dec 19;12:21904. doi: 10.1038/s41598-022-25542-2 (PMC9763421; doi:10.1038/s41598-022-25542-2)
Supplement: Supplementary file 1 — Supplementary Information. [file 41598_2022_25542_MOESM1_ESM.docx]

Special scattering regimes for conical all-dielectric nanoparticles

Alexey V. Kuznetsov^1,2,3,*^, Adrià Canós Valero^3,4^, Hadi K. Shamkhi^3,5^, Pavel Terekhov^6^, Xingjie Ni^6^, Vjaceslavs Bobrovs^2^, Mikhail V. Rybin^3^, Alexander S. Shalin^1,2,7,8,9*^

^1^Moscow Institute of Physics and Technology, Center for Photonics and 2D Materials, Dolgoprudny, 141700, Russia

^2^Riga Technical University, Institute of Telecommunications, Riga, 1048, Latvia

^3^ITMO University, Faculty of Physics, St. Petersburg, 197101, Russia

^4^University of Graz, and NAWI Graz, Institute of Physics, Graz, 8010, Austria

^5^A*STAR (Agency for Science, Technology and Research), Institute of Materials Research and Engineering, 138634, Singapore

^6^The Pennsylvania State University, Department of Electrical Engineering, Pennsylvania, 16802, United States

^7^Moscow State University, Faculty of Physics, Moscow, 119991, Russia

^8^Suzhou City University, School of Optical and Electronic Information, Suzhou, 215104, China

^9^Kotelnikov Institute of Radio Engineering and Electronics, Ulyanovsk, 432000, Russia

[*alexey.kuznetsov98@gmail.com](mailto:*alexey.kuznetsov98@gmail.com)

[*alexandesh@gmail.com](mailto:*alexandesh@gmail.com%20)

S1. Experimental Dispersion of amorphous Si


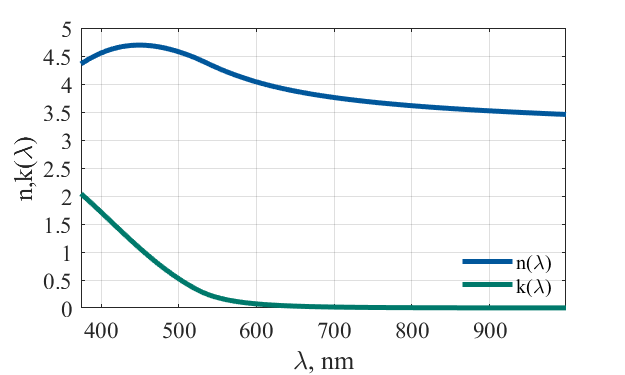


**Supplementary Figure S1.** Real (n) and imaginary (k) parts of the experimentally measured refractive index of amorphous silicon (aSi) employed in our study, as a function of wavelength.

S2. Conditions for Generalized Kerker effects

In the main part of the article the conditions for the forward Kerker effect were obtained. We used formula (6) in main part of manuscript and vanished the terms that do not participate in the effect.

ED + MD: $C_{sca}^{total}=C_{sca}^{p}+C_{sca}^{m}=\frac{k^{4}}{6\pi\varepsilon_{0}^{2}\left|\mathbf{E}_{inc}\right|^{2}}\left[\left|p_{x}\right|^{2}+\left|\frac{m_{y}}{c}\right|^{2}\right]$ (S1)

ED + EQ: $C_{sca}^{total}=C_{sca}^{p}+C_{sca}^{Q^{e}}=\frac{k^{4}}{6\pi\varepsilon_{0}^{2}\left|\mathbf{E}_{inc}\right|^{2}}\left[\left|p_{x}\right|^{2}+\frac{1}{120}\left(\left|kQ_{xz}^{e}\right|^{2}+\left|kQ_{zx}^{e}\right|^{2}\right)\right]$ (S2)

MD + MQ: $C_{sca}^{total}=C_{sca}^{m}+C_{sca}^{Q^{m}}=\frac{k^{4}}{6\pi\varepsilon_{0}^{2}\left|\mathbf{E}_{inc}\right|^{2}}\left[\left|\frac{m_{y}}{c}\right|^{2}+\frac{1}{120}\left(\left|\frac{kQ_{yz}^{m}}{c}\right|^{2}+\left|\frac{kQ_{zy}^{m}}{c}\right|^{2}\right)\right]$ (S3)

EQ + MQ: $C_{sca}^{total}=C_{sca}^{Q^{e}}+C_{sca}^{Q^{m}}=\frac{k^{4}}{6\pi\varepsilon_{0}^{2}\left|\mathbf{E}_{inc}\right|^{2}}\left[\frac{1}{120}\left(\left|kQ_{xz}^{e}\right|^{2}+\left|kQ_{zx}^{e}\right|^{2}+\left|\frac{kQ_{yz}^{m}}{c}\right|^{2}+\left|\frac{kQ_{zy}^{m}}{c}\right|^{2}\right)\right]$ (S4)

We proceed in a similar way with the formula (9) in main part of manuscript for the scattering cross-section of multipoles (n_z_ = -1, $E_{x}^{bwsc}=0$, because forward scattering only).

ED + MD: $E_{x}^{bwsc}=\frac{k^{2}}{4\pi\varepsilon_{0}}\frac{e^{ikr}}{r}\left\{p_{x}-\frac{1}{c}m_{y}\right\}=0$ (S5)

ED + EQ: $E_{x}^{bwsc}=\frac{k^{2}}{4\pi\varepsilon_{0}}\frac{e^{ikr}}{r}\left\{p_{x}+\frac{ik}{6}Q_{xz}^{e}\right\}=0$ (S6)

MD + MQ: $E_{x}^{bwsc}=\frac{k^{2}}{4\pi\varepsilon_{0}}\frac{e^{ikr}}{r}\left\{-\frac{1}{c}m_{y}-\frac{ik}{6c}Q_{yz}^{m}\right\}=0$ (S7)

EQ + MQ: $E_{x}^{bwsc}=\frac{k^{2}}{4\pi\varepsilon_{0}}\frac{e^{ikr}}{r}\left\{\frac{ik}{6}Q_{xz}^{e}-\frac{ik}{6c}Q_{yz}^{m}\right\}=0$ (S8)

Now we can take the multipoles moments from the formulas and substitute them into the formulas for the scattering cross-section to obtain conditions in the absence of backscattering.

ED + MD: $C_{sca}^{total}=C_{sca}^{p}+C_{sca}^{m}=\frac{k^{4}}{6\pi\varepsilon_{0}^{2}\left|\mathbf{E}_{inc}\right|^{2}}\left[\left|\frac{m_{y}}{c}\right|^{2}+\left|\frac{m_{y}}{c}\right|^{2}\right]$ (S9)

ED + EQ: $C_{sca}^{total}=C_{sca}^{p}+C_{sca}^{Q^{e}}=\frac{k^{4}}{6\pi\varepsilon_{0}^{2}\left|\mathbf{E}_{inc}\right|^{2}}\left[\frac{1}{36}\left|-ikQ_{xz}^{e}\right|^{2}+\frac{1}{60}\left(\left|kQ_{xz}^{e}\right|^{2}\right)\right]$ (S10)

MD + MQ: $C_{sca}^{total}=C_{sca}^{m}+C_{sca}^{Q^{m}}=\frac{k^{4}}{6\pi\varepsilon_{0}^{2}\left|\mathbf{E}_{inc}\right|^{2}}\left[\frac{1}{36}\left|-\frac{ikQ_{yz}^{m}}{c}\right|^{2}+\frac{1}{60}\left(\left|\frac{kQ_{yz}^{m}}{c}\right|^{2}\right)\right]$ (S11)

EQ + MQ: $C_{sca}^{total}=C_{sca}^{Q^{e}}+C_{sca}^{Q^{m}}=\frac{k^{4}}{6\pi\varepsilon_{0}^{2}\left|\mathbf{E}_{inc}\right|^{2}}\left[\frac{1}{60}\left(\left|\frac{kQ_{yz}^{m}}{c}\right|^{2}+\left|\frac{kQ_{yz}^{m}}{c}\right|^{2}\right)\right]$ (S12)

This shows how the scattering cross-sections of different multipoles must be related to each other to fulfill the conditions for the absence of backscattering. Similar calculations can be used to obtain the same values for the absence of forward scattering.

ED + MD: $\frac{C_{sca}^{ED}}{C_{sca}^{MD}}=1$ (S13)

ED + EQ: $\frac{C_{sca}^{ED}}{C_{sca}^{EQ}}=1.67$ (S14)

MD + MQ: $\frac{C_{sca}^{MD}}{C_{sca}^{MQ}}=1.67$ (S15)

EQ + MQ: $\frac{C_{sca}^{EQ}}{C_{sca}^{MQ}}=1$ (S16)

Following the same logic as for the Generalized Kerker effect, we obtain the conditions for the Transverse Kerker effect.

ED + MQ: $E_{x}^{bwsc}=E_{x}^{fwsc}=\frac{k^{2}}{4\pi\varepsilon_{0}}\frac{e^{ikr}}{r}\left\{p_{x}-\frac{ik}{6c}Q_{yz}^{m}\right\}=0$ (S17)

MD + EQ: $\left\{\begin{matrix} E_{x}^{bwsc}=\frac{k^{2}}{4\pi\varepsilon_{0}}\frac{e^{ikr}}{r}\left\{-\frac{1}{c}m_{y}+\frac{ik}{6}Q_{xz}^{e}\right\}=0 \\ E_{x}^{fwsc}=\frac{k^{2}}{4\pi\varepsilon_{0}}\frac{e^{ikr}}{r}\left\{\frac{1}{c}m_{y}-\frac{ik}{6}Q_{xz}^{e}\right\}=0 \end{matrix}\right.$ (S18)

Then

ED + MQ: $C_{sca}^{total}=C_{sca}^{p}+C_{sca}^{Q^{m}}=\frac{k^{4}}{6\pi\varepsilon_{0}^{2}\left|\mathbf{E}_{inc}\right|^{2}}\left[\frac{1}{36}\left|\frac{ikQ_{yz}^{m}}{c}\right|^{2}+\frac{1}{60}\left(\left|\frac{kQ_{yz}^{m}}{c}\right|^{2}\right)\right]$ (S19)

MD + EQ: $C_{sca}^{total}=C_{sca}^{m}+C_{sca}^{Q^{e}}=\frac{k^{4}}{6\pi\varepsilon_{0}^{2}\left|\mathbf{E}_{inc}\right|^{2}}\left[\frac{1}{36}\left|-ikQ_{xz}^{e}\right|^{2}+\frac{1}{60}\left(\left|kQ_{xz}^{e}\right|^{2}\right)\right]$ (S20)

Whence it follows that:

ED + MQ: $\frac{C_{sca}^{ED}}{C_{sca}^{MQ}}=1.67$ (S21)

MD + EQ: $\frac{C_{sca}^{MD}}{C_{sca}^{EQ}}=1.67$ (S22)

S3. Specific parameters of the considered effects for truncated silicon nanocones.

These data will make it easy to get any listed effect at the desired wavelength using the following simple relationships, provided that the refractive indices at these wavelengths are approximately equal:

$\left\{\begin{matrix} \frac{R_{top}}{R_{bottom}}n\approx\frac{R_{top}^{*}}{R_{bottom}^{*}}n^{*}\approx const \\ \begin{matrix} \frac{H}{\lambda}n\approx\frac{H^{*}}{\lambda^{*}}n^{*}\approx const \\ \frac{R}{\lambda}n\approx\frac{R^{*}}{\lambda^{*}}n^{*}\approx const \end{matrix} \end{matrix}\right.$ (S23)

, where the parameters without an asterisk are the parameters below and the parameters with an asterisk are the parameters to be retrieved.

|  | **Generalized** | **Transverse** |
| --- | --- | --- |
| **ED + MD** | H = 140 nm  R_top_ = 120 nm  R_bottom_ = 90 nm  λ = 779 nm | - |
| **ED + EQ** | H = 160 nm  R_top_ = 100 nm  R_bottom_ = 250 nm  λ = 675 nm | - |
| **ED + MQ** | - | H = 420 nm  R_top_ = 130 nm  R_bottom_ = 110 nm  λ = 779 nm |
| **MD + EQ** | - | H = 300 nm  R_top_ = 40 nm  R_bottom_ = 120 nm  λ = 617 nm |
| **MD + MQ** | H = 100 nm  R_top_ = 190 nm  R_bottom_ = 140 nm  λ = 767 nm | - |
| **EQ + MQ** | H = 320 nm  R_top_ = 360 nm  R_bottom_ = 180 nm  λ = 815 nm | - |
| **ED + MD + EQ + MQ** | H = 220 nm  R_top_ = 50 nm  R_bottom_ = 330 nm  λ = 625 nm | H = 520 nm  R_top_ = 55 nm  R_bottom_ = 195 nm  λ = 900 nm |

**Supplementary Table S1.** Specific parameters of nano-scatterers for obtaining Kerker effects for truncated silicon nanocones.

**Hybrid Anapole regime**

H = 200 nm, R_top_ = 40 nm, R_bottom_ = 340 nm, λ = 625 nm (truncated cone)

H = 369 nm, R = 127 nm, λ = 748 nm (cylinder)


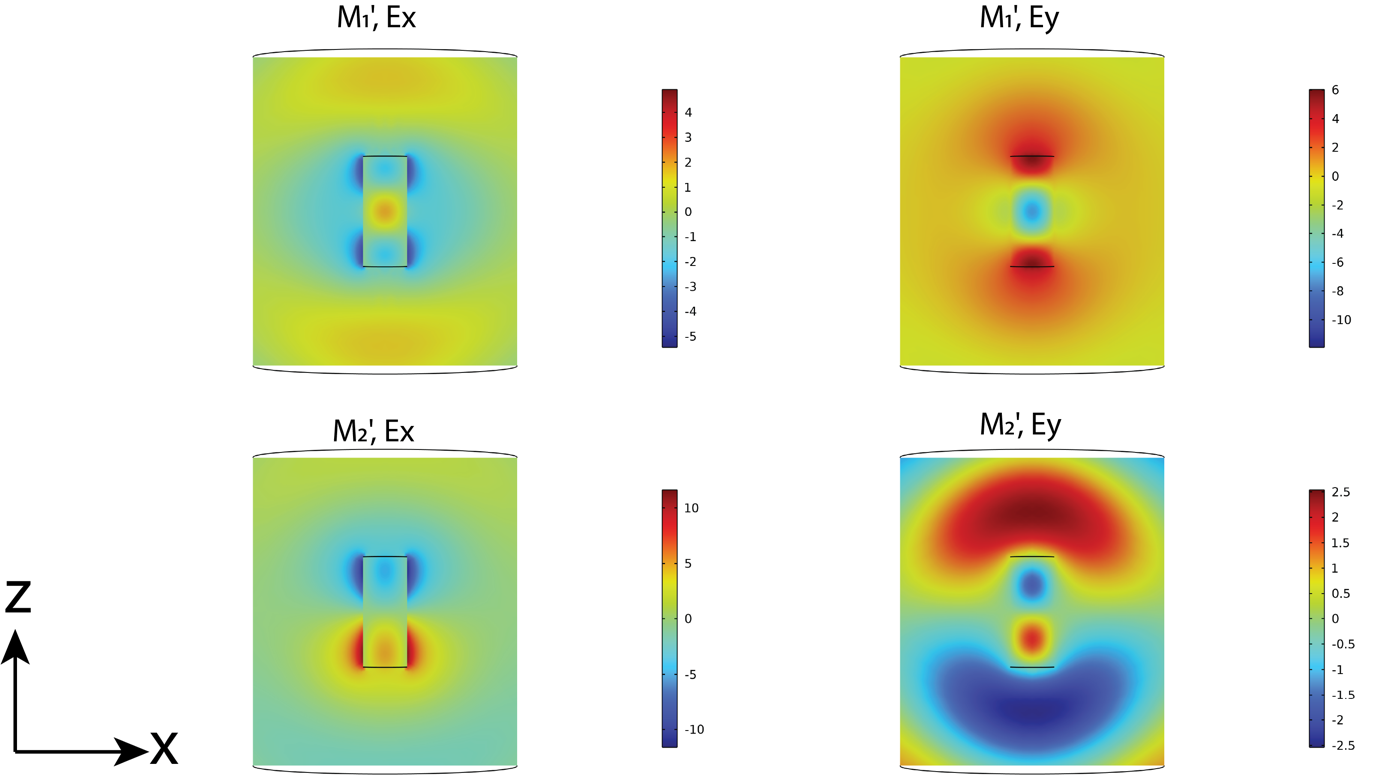


**Supplementary Figure S2.** QNMs M_1_' and M_2_' field distributions for the cylinder presented in figure 10 in the main text. The field distributions show M_1_' is an even-parity mode while M_2_' is an odd-parity mode.
